# Assessment of Indoor Air Pollution in Homes with Infants

**DOI:** 10.3390/ijerph8124502

**Published:** 2011-12-05

**Authors:** Anna Ruth Pickett, Michelle L. Bell

**Affiliations:** School of Forestry and Environmental Studies, Yale University, 195 Prospect St., New Haven, CT 06511, USA; Email: aruthpickett@gmail.com

**Keywords:** indoor air, infants, nurseries, carbon dioxide, carbon monoxide, volatile organic compounds, particulate matter

## Abstract

Infants spend most of their indoor time at home; however, residential air quality is poorly understood. We investigated the air quality of infants’ homes in the New England area of the U.S. Participants (*N* = 53) were parents of infants (0–6 months) who completed telephone surveys to identify potential pollutant sources in their residence. Carbon monoxide (CO), carbon dioxide (CO_2_), particulate matter with aerodynamic diameter ≤0.5 µm (PM_0.5_), and total volatile organic compounds (TVOCs) were measured in 10 homes over 4–7 days, and levels were compared with health-based guidelines. Pollutant levels varied substantially across homes and within homes with overall levels for some homes up to 20 times higher than for other homes. Average levels were 0.85 ppm, 663.2 ppm, 18.7 µg/m^3^, and 1626 µg/m^3^ for CO, CO_2_, PM_0.5_, and TVOCs, respectively. CO_2_, TVOCs, and PM_0.5_ levels exceeded health-based indoor air quality guidelines. Survey results suggest that nursery renovations and related potential pollutant sources may be associated with differences in urbanicity, income, and presence of older children with respiratory ailments, which could potentially confound health studies. While there are no standards for indoor residential air quality, our findings suggest that additional research is needed to assess indoor pollution exposure for infants, which may be a vulnerable population.

## 1. Introduction

Residential indoor air quality is not regulated, and the levels of indoor pollution are not widely known. Some sources of indoor air pollution in homes are solvents used in cleaning, building materials, paint, radon, allergens, cooking, smoking, plastics, carpets, and biomass burning for fuel or cooking [1,2,3,4,5]. Levels are affected by trends in building design and construction practices, such as reduced ventilation rates, more tightly sealed buildings, and synthetic building materials and furnishings. Solvents involved in renovations and painting in homes have been associated with increased risk of general respiratory symptoms for children under 5 years [1]. Volatile organic compounds (VOCs) are found in sources such as paints, furnishings, carpets, and household cleaning products. Many can be respiratory and sensory irritants, carcinogens, developmental toxins, neurotoxins, hepatotoxins, and immunosuppressants, and may cause symptoms that manifest as sick building syndrome [6].

Most people in the U.S. spend 90% or more of their time indoors [7]. A recent review of scientific literature on indoor air identified the study of how indoor air affects health as one of the greatest research needs [8]. The health effects of indoor air pollutants are not fully understood, but indoor air quality has been linked with a wide array of health outcomes including deficits in lung function, chronic respiratory disease, lung cancer, heart disease, developmental disorders, and damage to the brain, nervous system, liver, or kidneys [9,10,11,12]. Health consequences from indoor air can result from cumulative exposure possibly starting in infancy [13]. 

Children’s health outcomes have been associated with exposure to hazardous chemicals, many of which are present indoors. These health impacts include asthma, behavioral disorders, learning disabilities, autism, cancer, dysfunctional immune systems, neurological impairments and reproductive disorders [14]. A review of studies on air pollution exposure and sudden infant death syndrome (SIDS) concluded that while more research is needed, there exists suggestive evidence that air pollution affects SIDS [15,16]. The authors recommended further research on indoor air quality.

Infants are a unique and important subpopulation to study with respect to indoor air pollution for several reasons. They spend a majority of their time indoors [17]. Their exposures can deliver higher doses as infants breathe more air per body weight than adults. Their respiratory and other systems are under development. Mouth breathing, which bypasses the filter of the nose, is more common in infants than adults. Mouth breathing may pull air pollutants deeper into the respiratory system, which could result in a different composition of the pollutant mixture at the alveolar level [18]. Homes of newborn infants are of special interest because parents may consider renovation and redecoration that impacts indoor air quality, such as through indoor painting. 

Little research has been conducted on infant exposures to indoor air pollution and consequent health outcomes, partially due to the lack of available monitoring data analogous to that for ambient pollution, the heterogeneity in exposures across homes, ethical considerations regarding human exposure studies for infants, and the challenges of using animal models due to the animal and human differences of gestation periods and developmental stages at birth [19]. To help address the significant gap in the scientific literature on infants’ exposure to indoor air pollution, we performed a study of indoor air pollution in homes of newborn infants in the New England area of the U.S. using monitoring data to quantify exposures and survey data to assess potential sources of exposures.

## 2. Experimental Section

### 2.1. Recruitment

Subjects were required to live in the northeastern U.S. and have an infant age 0–6 months at the time of enrollment. No other exclusion criteria were applied. Recruitment began June 2009 and continued until October 2009 and was conducted through distribution of flyers in thirteen maternity clinics, hospitals, and birthing classes throughout the northeastern U.S. Consent was obtained from the places of business where the flyers were posted. Advertisements were posted online on new mother websites and community billboards. In exchange for participation in the phone survey, each participant was offered $20 to be paid after the survey. At the conclusion of the study, all participants were offered results from the study and a brochure on indoor air quality. Subjects who participated in the air monitoring were offered the monitoring results for their home. The Yale Human Subjects Committee approved this study. The five stages of the study design were thus: recruitment; phone survey; indoor air monitoring; data analysis; and information distribution to study participants.

### 2.2. Indoor Air Quality Monitoring

Indoor air monitoring was conducted for carbon monoxide (CO), carbon dioxide (CO_2_), particulate matter with aerodynamic diameter ≤0.5 (PM_0.5_), total volatile organic compounds (TVOCs), temperature, and humidity. These pollutants were chosen based on previous observed health impacts and compatibility with available monitors [15,20,21,22,23,24,25,26]. Measurements were taken between June 19 and August 9, 2009 in ten homes: three homes in Connecticut, three in New York, two in Vermont, and two in Massachusetts. The air monitor was placed in the nursery approximately two feet above the floor on a horizontal surface (e.g., chair, shelf, table) away from windows and doors, and measurements were taken for 4 to 7 days in each home. For the purpose of this study, the term “nursery” is used to describe the area in the home where the infant spends the majority of his/her time, as identified by the participants. 

Monitoring was conducted with an AirAdvice M7100 [20,21]. TVOCs and PM_0.5_ are reported in µg/m^3^ and CO_2_ and CO in ppm. This monitor does not measure other forms of PM, such as particulate matter with aerodynamic diameter ≤2.5 µm or ≤10 µm (PM_2.5_ or PM_10_). For measurements that exceeded the CO_2_ monitor limit of 2000 ppm, summary measures (e.g., overall average of CO_2_ in a home during the monitoring period) were calculated with these values as 2000 ppm, which underestimates the true pollution levels. The pollutant measurements were analyzed to determine the variability and overall levels of each pollutant within the nurseries and among the participants. Variability was assessed by summary statistics, boxplots, analysis of variance, and coefficient of variance (COV), which is the standard deviation divided by the mean ×100%, with larger values indicating higher variability in relation to the mean value.

### 2.3. Survey of Potential Indoor Pollutant Sources

A telephone survey of 53 study subjects was conducted to assess potential sources of residential indoor air pollution, and to investigate the degree to which the arrival of a new infant coincided with activities that may relate to indoor air pollution (e.g., indoor painting). The 10 subjects who participated in air pollution monitoring also participated in the survey, and were randomly selected. Questions elicited information on the parent characteristics (e.g., mother’s education) and household characteristics (e.g., type of heating fuel). The survey focused on aspects of the home that may impact air quality, such as renovation prior to or shortly after the infant’s arrival, air purifiers, and presence of cockroaches [27,28,29,30,31,32]. Questions pertaining to renovation refer to activities taking place in the last six months of pregnancy to the time of the survey. All surveys were conducted by a single investigator to ensure consistency.

### 2.4. Dissemination of Results to Participants

At the conclusion of the study, each participant was offered the results from the study for overall findings and a U.S. Environmental Protection Agency (U.S. EPA) brochure providing general information on indoor air quality [33]. Participants in the monitoring phase of the study were offered monitoring results for their own home. 

## 3. Results and Discussion

### 3.1. Indoor Air Quality Monitoring Results

Monitoring for indoor air quality was conducted in the room in which the infant spends the most time for 10 homes. Table 1 summarizes the pollutant levels, temperature, and humidity in each home. Pollutant levels varied greatly across homes with overall levels for some homes up to 20 times higher than for another home (e.g., TVOCs for Participants D and E). The homes with the highest levels varied by pollutant (Participants F and I for CO, D and I for CO_2_, B and C for PM_0.5_, and D and F for TVOCs). Those with the highest overall levels were not necessarily those with the highest peak levels. For instance, Participant A’s average PM_0.5_ level was lower than that of most other homes (6 of 9), but had the highest hourly value. 

**Figure 1:**
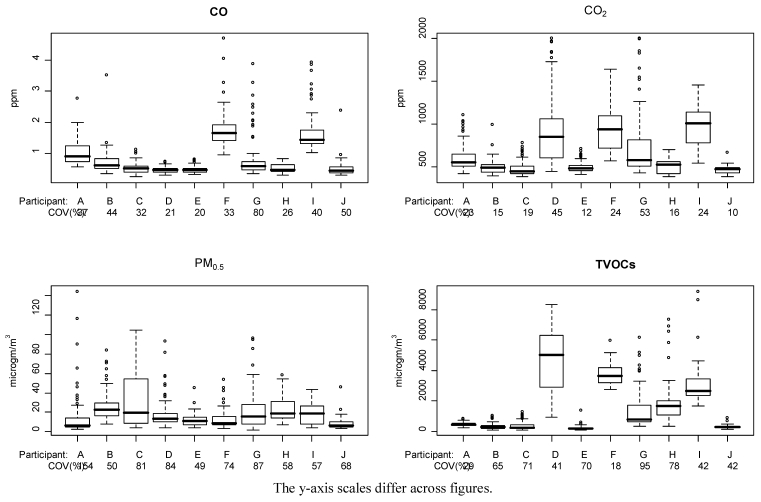
Boxplots of pollutant measurements with COV, by pollutant and home.

Figure 1 provides boxplots of hourly pollutant levels for each pollutant and home, and illustrates the variability of pollutant levels within a given nursery and across homes. Ninety percent of hourly measurements were between 0.37 and 1.93 ppm for CO, 411.9 and 1194 ppm for CO_2_, 4.4 and 53.3 µg/m^3^ for PM_0.5_, and 124.0 and 5171 μg/m^3^ for TVOCs. Figure 1 also provides the COV for each pollutant and home. Participant G had the highest variability, based on the COV, for CO, CO_2_, and TVOCs, and the second highest variability for PM_0.5_. CO_2_ exhibited the lowest average variability in relation to the mean and PM_0.5_ the highest. Between participant variability was larger than within participant variability for all pollutants, based on analysis of variance (*p*-value <0.001). TVOC levels appeared highly variable both across the nurseries and within individual nurseries (Figure 1d), with average levels within a home ranging from 210.8 to 4683 µg/m^3^ and some measurements exceeding 6000 µg/m^3^.

**Table 1:** Average, median (minimum to maximum) hourly value of air quality measurements in each nursery.

	CO [ppm]	CO_2_ [ppm]	PM_0.5_ [μg/m^3^]	TVOCs [μg/m^3^]	Temperature [°F]	Relative humidity [%]
Participant A	1.04, 0.92	603.7, 556.3	13.8, 6.2	459.6, 442.5	74.0, 74.1	64.6, 64.6
	(0.57 to 2.78)	(419.9 to 1102)	(2.6 to 143.9)	(241.2 to 811.4)	(68.3 to 78.6)	(60.5 to 68.0)
Participant B	0.71, 0.63	494.9, 487.4	25.0, 23.0	328.6, 285.5	79.3, 79.7	59.0, 59.6
	(0.35 to 3.52)	(396.8 to 994.4)	(8.3 to 83.4)	(95.2 to 104.8)	(72.4 to 83.6)	(44.2 to 72.7)
Participant C	0.52, 0.52	484.5, 450.1	32.2, 19.7	346.1, 242.9	80.5, 80.1	54.1, 57.3
	(0.25 to 1.13)	(382.3 to 784.4)	(4.2 to 104.1)	(105.8 to 1253)	(77.2 to 83.9)	(37.0 to 69.3)
Participant D	0.48, 0.47	934.6, 847.0	18.4, 13.7	4683, 5031	76.1, 76.7	60.8, 61.0
	(0.30 to 0.74)	(445.2 to 1999)	4.5 to 92.6)	(993.4 to 8321)	(72.1 to 80.0)	(49.1 to 67.0)
Participant E	0.49, 0.47	491.5, 480.1	11.7, 11.0	210.8, 168.7	77.0, 77.1	53.4, 54.9
	(0.34 to 0.81)	(415.3 to 709.7)	(3.9 to 45.4)	(89.2 to 1351)	(72.1 to 80.4)	(40.4 to 64.4)
Participant F	1.76, 1.65	932.7, 935.9	12.6, 8.79	3722, 3627	75.4, 75.4	60.7, 60.0
	(0.97 to 4.69)	(573.5 to 1638)	(3.5 to 53.6)	(2730 to 5941)	(72.6 to 76.2)	(51.2 to 68.1)
Participant G	0.79, 0.59	745.3, 582.7	21.8, 15.6	1343, 791.8	74.4, 74.5	68.0, 67.6
	(0.35 to 3.88)	(433.3 to >2000)	(2.0 to 95.7)	(382.8 to 6171)	(67.8 to 79.5)	(61.3 to 74.1)
Participant H	0.53, 0.48	506.0, 523.2	23.6, 18.9	1862, 1660	71.3, 71.3	72.1, 70.9
	(0.32 to 0.85)	(382.5 to 703.3)	(7.5 to 57.8)	(327.2 to 7363)	(68.1 to 77.5)	(65.0 to 80.0)
Participant I	1.70, 1.45	974.1, 1011	19.1, 19.1	3020, 2635	72.1, 72.2	53.3, 52.5
	(1.04 to 3.94)	(544.0 to 1458)	(3.94 to 43.2)	(1658 to 9168)	(69.7 to 74.4)	(47.9 to 60.6)
Participant J	0.51, 0.44	464.2, 471.0	8.7, 6.7	287.2, 60.0	74.0, 74.6	59.8, 60.3
	(0.30 to 2.37)	(382.1 to 671.1)	(3.2 to 46.0)	(117.4 to 858.7)	(67.8 to 79.0)	(46.9 to 71.3)
All participants ^1^	0.85, 0.79	663.2, 646.8	18.7, 13.8	1626, 1498	75.4, 76.0	60.5, 59.8
	(0.25 to 4.69)	(382.1 to >2000)	(2.0 to 143.9)	(89.2 to 9186)	(70.8 to 79.3)	(50.4 to 69.6)

^1^ The number of measurements varied by participant. Averages across all participants represent the average of each participant’s overall average. The minimum and maximum for all participants represent the lowest and highest values recorded for any participant.

We calculated Pearson’s correlations among all pollutant pairs for each home (Table 2). The highest correlations were among TVOC and CO_2_ although this relationship varied across homes (average correlation 0.68, range 0.42 to 0.94). Other high correlations were noted for CO and CO_2_ (average 0.60, range 0.06 to 0.90). Table 2 also shows the correlations between pollutant levels and weather variables. On average across the 10 homes, pollutants were not correlated with temperature or relative humidity, although one home had a correlation of 0.71 for humidity and CO_2_, and another of 0.81 for humidity and PM_0.5_.

**Table 2:** Average (minimum to maximum) of within home pollutant correlations and weather variables.

	CO_2_	PM_0.5_	TVOC	Temperature	Relative humidity
**CO**	0.60	0.20	0.42	0.08	0.14
	(0.06 to 0.90)	(−0.27 to 0.48)	(−0.14 to 0.83)	(−0.19 to 0.35)	(−0.34 to 0.49)
**CO_2_**		0.11	0.68	−0.02	0.25
		(−0.70 to 0.66)	(0.42 to 0.94)	(−0.49 to 0.58)	(−0.23 to 0.71)
**PM_0.5_**			0.13	0.01	0.29
			(−0.17 to 0.71)	(−0.59 to 0.39)	(−0.22 to 0.81)
**TVOC**				0.01	0.11
				(−0.35 to 0.50)	(−0.35 to 0.52)
**Temperature**					−0.08
					(−0.65 to 0.89)

*Note:* The minimum and maximum represent the smallest and largest correlations within a given home.

**Figure 2:**
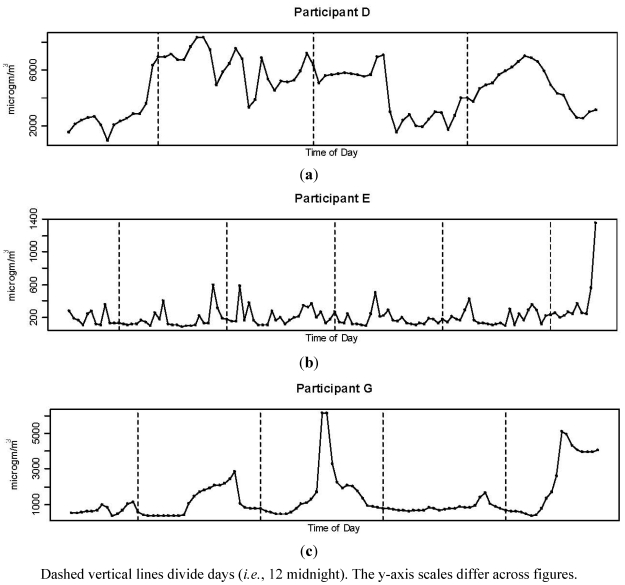
Hourly average TVOC across time for three participants: (**a**) Participant D; (**b**) Participante E; (**c**) Participant G.

Results indicate that the daily pattern of exposure to indoor air pollution in nurseries also differs across homes. As an example, Figure 2 shows hourly average levels of TVOC concentrations across time in three homes. Levels in Participant D’s home averaged 4683 μg/m^3^ and exceeded 6000 μg/m^3^ at some point each day. Levels for Participant E followed a different pattern, averaging 210.8 μg/m^3^ and below 500 μg/m^3^ for most of the measurement period, then rising sharply to an hourly average >1,300 μg/m^3^ during the final day.

TVOCs in Participant G’s home averaged 1343 μg/m^3^ and varied widely with full-day (24-h) daily averages ranging from 832 to 1638 μg/m^3^ and hourly averages >5000 μg/m^3^ on two days. Supplemental Figures 1–4 provide similar graphs for all four pollutants and homes.

### 3.2. Comparison of Monitoring Results to Air Quality Guidelines

Indoor air quality in residences is not regulated; however for context, we compared the measured levels to several air quality guidelines and standards including health-based regulations for outdoor air, regulations for indoor air in occupational settings, and indoor air quality guidelines. Table 3 provides air quality guidelines developed by AirAdvice using information from the U.S. EPA; the American Society of Heating, Refrigerating and Air-Conditioning Engineers (ASHRAE); World Health Organization (WHO); Leadership in Environmental and Ecological Design (LEED); Indoor Air Quality Association; California Air Resources Board; Health Canada; European Union; U.K. Department of Health; state governments; and scientific experts [20,21]. Table 4 provides the air quality metrics for each pollutant based on the exposure timeframe used in Table 3 (*i.e.*, daily average for PM_0.5_, CO_2_, and TVOCs; maximum 1-h and 8-h average for CO) for each participant and each day (Table 4). We also compared measurements to air quality guidelines developed by the Occupational Safety and Health Administration (OSHA), the American Conference of Governmental Industrial Hygienists (ACGIH), and the National Institute for Occupational Safety and Health (NIOSH) for indoor air in occupational settings. Other comparisons include guidelines from the Consumer Product Safety Commission, residential air quality guidelines from Health Canada, and U.S. EPA health-based regulations and WHO guidelines for outdoor air.

**Table 3:** Indoor air quality guidelines (based on [20,21]).

CO	CO_2_	PM_0.5_	TVOCs	Indoor Air Quality Rating
<5 ppm, 8-h average	≤750 ppm, daily average	≤10 μg/m^3^, daily average	≤500 μg/m^3^, daily average	Acceptable
5–9 ppm, 8-h average	751–999 ppm, daily average	11–25 μg/m^3^, daily average	501–3000 μg/m^3^, daily average	Action recommended for sensitive groups ^1^
		26–35 μg/m^3^, daily average		Action recommended
>20 ppm, 1-h average and/or >9 ppm, 8-h average	>999 ppm, daily average	>35 μg/m^3^, daily average	>3000 μg/m^3^, daily average	Action necessary

^1^ Sensitive groups are defined as: “the elderly; children under the age of 18; asthma and allergy sufferers; pregnant women and their unborn children; immuno-compromised individuals; and those with cardiovascular, respiratory, and other chronic disease conditions” [20,21].

**Table 4:** Pollutant levels for each participant and day.

**a. CO daily 1-h maximum (ppm)**								
Participant	Day 1	Day 2	Day 3	Day 4	Day 5	Day 6	Day 7	Day 8
A	2.0	1.5	2.8	0.9	0.9			
B	3.5	1.2	1.0	0.9	0.8	1.0	1.1	0.6
C	0.6	0.6	0.6	1.1	0.6			
D	0.5	0.7	0.6	0.7				
E	0.8	0.6	0.6	0.6	0.6	0.4		
F	4.7	2.2	2.0	2.3				
G	0.7	0.8	2.0	1.5	3.9			
H	0.7	0.7	0.5	0.8				
I	1.9	1.8	3.9	1.5				
J	1.0	0.6	2.4	0.7				
**b. CO2 daily average (ppm)**								
Participant	Day 1	Day 2	Day 3	Day 4	Day 5	Day 6	Day 7	Day 8
A	763	555	653	538	528			
B	511	519	487	481	482	497	483	483
C	475	456	443	525	517			
D	872	1111	765	973				
E	496	485	468	474	479	629		
F	1148	965	872	841				
G	575	712	697	557	1238			
H	509	482	477	582				
I	1040	1057	1059	767				
J	469	444	467	492				
**c. PM0.5 daily average (μg/m^3^)**								
Participant	Day 1	Day 2	Day 3	Day 4	Day 5	Day 6	Day 7	Day 8
A	5.8	13.1	20.3	9.1	21.7			
B	23.9	30.3	19.0	22.1	28.3	20.4	27.2	35.3
C	7.2	9.0	29.7	57.1	36.6			
D	20.8	16.4	18.7	18.7				
E	14.4	10.8	8.3	13.4	14.6	7.9		
F	8.3	9.1	16.5	14.7				
G	16.4	15.7	38.9	16.0	19.3			
H	23.2	37.2	17.3	13.1				
I	11.2	15.5	18.5	25.9				
J	14.7	7.8	7.1	6.4				
**d. TVOC daily average (μg/m^3^)**								
Participant	Day 1	Day 2	Day 3	Day 4	Day 5	Day 6	Day 7	Day 8
A	771	520	811	749	570			
B	1000	933	584	512	1042	644	463	348
C	265	514	509	937	1253			
D	6919	8321	7048	6953				
E	360	593	586	504	428	1351		
F	4270	5148	5941	3753				
G	1148	2843	6171	1688	5148			
H	678	3042	2390	7363				
I	2009	9186	4613	3095				
J	483	476	363	859				

Each day is color coded corresponding to the indoor air quality guidelines from Table 3.

The daily maximum 1-h CO level for each day and nursery are provided in Table 4a. CO levels for all nurseries met the guidelines in Table 3 with all hourly values <20 ppm and 8-hour values <5 ppm. Measurements of CO met the Consumer Product Safety Commission recommended maximum levels of 15 ppm for 1 h and 25 ppm for 8 h, and the Health Canada recommendations for residential air quality at 25 ppm for 1 h and 10 ppm for 24 h [34]. The measured levels also were well within standards and guidelines for occupational settings: the OSHA permissible exposure limit (PEL) for general industry of 50 ppm for an 8-h exposure [35], the NIOSH recommended exposure limit (REL) of 35 ppm 8-h average based on cardiovascular response [36], and ACGIH threshold limit value of 25 ppm 8-h average based on risk of elevated carboxyhemoglobin levels [37]. Measured CO was below EPA’s National Ambient Air Quality Standards of 9 ppm for 8 h and 35 ppm for 1 h [38] and the WHO’s guidelines for ambient air for 10 ppm for 8-h and 25 ppm for 1 h [39]. 

Table 4b compares daily CO_2_ levels for a given nursery to air quality guidelines. For four nurseries at least one daily CO_2_ level exceeded the guideline for action needed, >999 ppm as per Table 3, and for one of these homes, that level was reached three out of four days measured. An additional household had one daily average at a level for recommended action for sensitive individuals. For four of the homes, CO_2_ levels exceeded ASHRAE recommendations that indoor levels be ≤1000 ppm for schools and ≤800 ppm for offices [40]. Recommendations for occupational settings are much higher at NIOSH guidelines of 5000 ppm for an 8-h exposure and 30,000 ppm for any 10-min period, OSHA PEL of 5000 ppm for an 8-h period [35]. Levels were also well below ACGIH guidelines of 5000 ppm for an 8-h exposure and 30,000 ppm for a short-term exposure limit [41]. Based on AirAdvice criteria, all homes had PM_0.5_ and TVOC levels above the threshold for action recommended for sensitive groups (Table 4c,d). The levels for action necessary were exceeded by three homes for PM_0.5_ and half of the homes for TVOCs.

### 3.3. Survey Results

Supplemental Table 1 describes the participants in the telephone survey (*N* = 53). Most respondents were mothers, with average age of 31.3 years (range 19–43 years) and infants with an average age of 97 days (range 6–192 days). No participants were pregnant at the time of the survey. Participants were predominately white (85%). The subjects’ educational levels were high with all mothers having completed at least one year of college, and almost 19% with 20 or more years of school. More than 79% of participant households had annual incomes exceeding $50,000. Of the 25% of homes with older siblings, 15% of those siblings had asthma and 38% had allergies. The infants spent most of their time in the home, with 9.4% in daycare (average 36 h/week). 

Table 5 describes housing characteristics for the study participants. Most houses were older (average year built was around the early 1940s, range 1810–2005). The most common home heating sources were oil, gas, and electric, with only 5.7% for wood, and some homes having multiple heating sources. Other potential sources of indoor air pollution identified by 20% or more of participants were fireplaces (21%), gas stoves (62%), attached garage (28%), mice (32%), and indoor pesticides (21%). Of the 51% with pets, 44% had cats and 63% had dogs. Aromatic candles were in the nursery for 15.1% of the nurseries. Of the candle users, 38% kept the candles unlit. Smoking took place in 3.8% of the homes. Survey participants estimated the distance between the home and the nearest road at an average of 63 feet, with 53% less than 25 feet away.

**Table 5:** Description of survey participants’ housing characteristics (*N* = 53), by monitoring group.

	Number of participants (%)		
	All participants (*N* = 53)	Monitoring participants (*n* = 10)	Non-monitoring participants (*n* = 43)
Neighborhood environment			
Urban	26 (49.1%)	6 (60%)	20 (46.5%)
Suburban	18 (34.0%)	3 (30%)	6 (14.0%)
Rural	9 (17.0%)	1 (10%)	17 (39.5%)
Home structure			
Single-family detached dwelling	19 (35.8%)	4 (40%)	15 (34.9%)
Townhouse or duplex	12 (22.6%)	1 (10%)	11 (25.6%)
Multiple story apartment building	22 (41.5%)	5 (50%)	17 (39.5%)
Year home built			
<1900	8 (15.1%)	1 (10.0%)	7 (16.3%)
1900–1949	19 (35.8%)	1 (10.0%)	18 (41.9%)
1950–2000	24 (45.3%)	7 (70.0%)	17 (39.5%)
>2000	2 (3.8%)	1 (10.0%)	1 (2.3%)
Distance to roadway (feet)			
<10	9 (17.0%)	2 (20.0%)	7 (16.3%)
10–25	19 (35.8%)	3 (30.0%)	16 (37.2%)
26–50	13 (24.5%)	1 (10.0%)	12 (27.9%)
51–200	9 (17.0%)	3 (30.0%)	6 (14.0%)
>200	3 (5.7%)	1 (10.0%)	2 (4.7%)
Heating source (homes may have multiple sources)			
Oil	16 (30.2%)	2 (20.0%)	14 (32.6%)
Gas	22 (41.5%)	5 (50.0%)	17 (39.5%)
Electric	13 (24.5%)	2 (20.0%)	11 (25.6%)
Wood	3 (5.7%)	4 (5.7%)	2 (4.7%)
Unknown	1 (1.9%)	0 (0%)	1 (2.3%)
Stove type			
Electric	19 (35.8%)	3 (30.0%)	16 (37.2%)
Gas	33 (62.3%)	7 (70.0%)	26 (60.5%)
Propane	1 (1.9%)	0 (0.0%)	1 (2.3%)
Presence in home			
Smoker living in home	2 (2.3%)	0 (10.0%)	2 (4.7%)
Smoking in home	2 (3.8%)	1 (10.0%)	1 (2.3%)
Fireplace (used)	11 (20.8%)	2 (20.0%)	9 (20.9%)
Attached garage	15 (28.3%)	5 (50.0%)	10 (23.3%)
Cockroaches	4 (7.5%)	1 (10.0%)	3 (7.0%)
Mice	17 (32.1%)	3 (30.0%)	14 (32.6%)
Pesticides	11 (20.8%)	1 (10.0%)	10 (23.3%)
Pets (any)	27 (50.9%)	5 (50.0%)	22 (51.2%)
Cats	12 (22.6%)	4 (40.0%)	8 (18.6%)
Dogs	17 (32.1%)	1 (10.0%)	16 (37.2%)
Plants	35 (66.0%)	8 (80.0%)	27 (62.8%)
Presence in nursery			
Room deodorizers	4 (7.5%)	1 (10.0%)	3 (7.0%)
Air purifiers	7 (13.2%)	1 (10.0%)	6 (14.0%)
Aromatic candles (lit)	5 (9.4%)	0 (0%)	2 (4.7%)
Aromatic candles (unlit)	3 (5.7%)	0 (0.0%)	6 (14.0%)

Table 6 describes renovation characteristics for the study participants. Of the 53 participants, 66% had remodeled the nursery at some point during the last six months of pregnancy to the time of the survey, with fewer (42%) having remodeled another portion of the house. Of those that remodeled the nursery, 11% of participants responded that renovations were complete before the infant’s arrival, on average 52 days prior to birth (range 24–92 days). Painting had taken place in the nursery for 49% of the participants, with interior walls painted more often than furniture, floor, or trim. Of those who painted, 46% reported using low-VOC paint. Area rugs were added to 28% of nurseries, 73% of which were new. Those who renovated the nursery did not differ from those who did not by mother’s demographics (age, race, or education) or the age of the home age. Similarly, those who painted the nursery and those who did not were similar on these factors (results not shown). 

**Table 6:** Description of survey participants’ renovations (*N* = 53), by monitoring group.

	Number of participants (%)		
	All participants (*N* = 53)	Monitoring participants (*n* = 10)	Non-monitoring participants (*n* = 43)
*Remodeled nursery*	35 (66.0%)	8 (80.0%)	27 (62.8%)
*Remodeled another part of house*	22 (41.5%)	5 (50.0%)	17 (39.5%)
*Renovations complete before birth*	4 (7.5%)	4 (40.0%)	0 (0.0%)
*Painting in nursery*	26 (49.1%)	4 (40.0%)	22 (51.2%)
Interior walls	13 (24.5%)	4 (40.0%)	9 (20.9%)
Furniture	3 (5.7%)	0 (0.0%)	3 (7.0%)
Floor	1 (1.9%)	0 (0.0%)	1 (2.3%)
Trim	4 (7.5%)	1 (10.0%)	2 (7.0%)
Low VOC paint	12 (22.6%)	3 (30.0%)	9 (20.9%)
Wallpaper	7 (13.2%)	2 (20.0%)	5 (11.6%)
Decals	2 (3.8%)	0 (0.0%)	2 (4.7%)
Used area rug	4 (7.5%)	1 (10.0%)	3 (7.0%)
New area rug	11 (20.8%)	2 (20.0%)	9 (20.9%)
Carpet	4 (7.5%)	1 (10.0%)	3 (7.0%)

Several general trends were observed in the survey data, although differences are not statistically significant and sample size is limited. Those in rural environments were more likely to renovate (89%) or paint (78%) the nursery than those in urban environments (6.7% renovated, 44% painted). Of those with household annual income <$40,000, 71% renovated the nursery and 75% lived in rural environments, compared to 55% and 0% of those with annual income >$100,000, respectively. A similar fraction of those with income <$40,000 painted the nursery (57%) as those with incomes >$100,000 (50%); however, painters in the lower income group were more likely to select low-VOC paint (75%) than painters in the higher income group (40%). Those with older children with asthma or allergies were less likely to have potential indoor air pollution sources than those without older children with these health concerns for: wallpaper installation in the nursery (0% *vs.* 15%), painting of the nursery (40% *vs.* 50%), use of indoor pesticides (0% *vs.* 23%), smoking in the home (0% *vs.* 4.2%), and room deodorizers in the nursery (0% *vs.* 8.3%). Those with older children with asthma or allergies were more likely to have air purifiers in the nursery (20% *vs.* 13%) and less likely to have pets (40% *vs.* 52%).

## 4. Conclusions

The main goal of this research is to expand the scientific literature on indoor air pollution, with a focus on infants’ exposure and source identification in the home. Little research has been conducted on residential indoor air environments for infants compared to the literature on ambient air, occupational indoor exposure, or other studies of exposure in other age groups. To date, most studies of infants and air pollution have been based on ambient pollution, with links to apnea and bradycardia [42] and infant mortality [43,44,45]. Several studies have investigated indoor air in homes for children. For example, Hulin *et al*. measured NO_2_, PM_2.5_, and VOCS in urban and rural homes in France for 51 children (mean age 12.6 years), finding associations with asthma and VOCs [46]. In Taiwan, the presence of mold in homes of children ages 4–7 years was not associated with biomarkers of allergic response [47]. Researchers have investigated air in other settings for children, including childcare centers. These studies include measures of phenols in North Carolina and Ohio [48]; radon, lead, asbestos, and mold in New York State [49]; CO_2_ in a Midwestern county in the U.S. [50]; and ozone in Singapore [51].

Studies have examined infants’ health in relation to indoor air pollution from biomass burning in India [52], Kenya [53], and Gambia [54]. One of the few studies to examine infants’ exposure in to indoor air pollution in homes in an industrialized country measured long-term exposure to NO*_x_*, NO_2_, formaldehyde, PM_2.5_, and black smoke in homes of 411 infants in Denmark, finding no association with risk of wheezing [55]. Raaschou-Nielsen *et al*. measured PM_2.5_ and black smoke in homes of 389 infants in Denmark, identifying a variety of sources, such as frying without a range hood, smoking, renovation, and local traffic [56]. Exposure to indoor pollutants of allergens (e.g., dust mite, cat, dog) and mold have been examined in relation to respiratory symptoms in infants [57,58]. A recent study of infants’ homes in Syracuse, New York, U.S., found that levels of PM_10_ and PM_2.5_ varied substantially across homes and within homes [59], which is consistent with our results. Higher levels of indoor PM_2.5_ was associated with infant wheeze. In that study, 68% of participants were smokers, compared to 2.3% of homes with smokers in our study [59].

The survey results show that in this limited sample, main sources of indoor pollutants may include renovation, which was conducted prior to or shortly after the infant’s arrival in 66.0% of homes. The potential factors affecting indoor air quality that were present in more than half the homes surveyed are gas stoves, pets, and remodeling of the nursery. General trends in survey results suggest that some populations may be more likely to conduct renovations of the nursery than others, potentially introducing sources of indoor air pollution such as through painting. This result indicates the potential for confounding if those populations (e.g., socio-economic conditions, urbanicity, existing health conditions in the family) are associated with the outcomes of interest in studies of infants’ health.

The limited sample size and narrow variation in participants’ demographics limit the generalizability of this study, as the population is mostly non-Hispanic white, highly educated, and high income. Participants who responded to the recruitment advertisements may have been drawn to volunteer for this study because they previously were interested in environmental issues and therefore may have taken more precautions to limit air pollution in their homes. Future efforts are needed with larger sample sizes to permit study of pollutant levels in relation to various household activities and characteristics, as well as different populations. For instance, our study population had a low reported rate of smoking in the home (3.8%), although environmental tobacco smoke is associated with a range of health outcomes for infants, such as low birth weight [60,61,62]. 

Research is needed to assess infants’ exposure to a more comprehensive set of indoor air pollutant measurements, such as biological contaminants including mold, dust mites, pet dander, pollen, dust, environmental tobacco smoke, other size fractions of particles (PM_2.5_, PM_10_), specific VOCs, dust, and radon. Data on home activities that could relate to indoor air quality could include detailed information on cooking, smoking, and cleaning. Future work could incorporate the penetration of ambient air pollution into the indoor environment, including differences by season, and information such as information on proximity to various types of roadways. Additional efforts are needed to better understand infants’ exposure to various pollutants in the indoor environment, including techniques to estimate levels with limited monitoring. For example, a microenvironment approach was developed for assessing infants’ exposure to indoor air pollution for respirable suspended particles and CO based on mobility patterns of infants and mothers [63]. Our findings indicate heterogeneity both within and across homes, suggesting the need for individual-level exposure assessments considering a wide range of settings, including different seasons, regions, demographics, and time-activity information that may affect pollutant levels (e.g., cooking, cleaning, opening windows). The time-activity data are particularly important to help understand the variation and large peaks that occur in pollutant concentrations throughout the day.

This study provides individual-level data that is illustrative of the ranges of pollutant levels that can be found in nurseries, with variation across homes and throughout the day within a single home. Although indoor air quality is not regulated, we observed levels of CO_2_, VOCs, and PM_0.5_ that exceeded health-based guidelines, indicating that residential air pollution may pose a health risk for infants. Given the limited sample size of this study, linking these exposure measurements to health outcomes is not appropriate. However, these results provide some of the first measurements of indoor air quality in homes of infants, and are valuable given the paucity of existing data. Understanding exposure assessment can be one of the first steps toward understanding how those exposures affect infants’ health.

## Supplementary Files

Supplementary File 1: DOC-Document (DOC, 378 KB)
