# Supplementary material for: Assessment of Indoor Air Pollution in Homes with Infants
Source: Int J Environ Res Public Health. 2011 Dec 5;8(12):4502–20. doi: 10.3390/ijerph8124502 (PMC3290986; doi:10.3390/ijerph8124502)
Supplement: Supplementary File 1: — DOC-Document (DOC, 378 KB) [file ijerph-08-04502-s001.doc]

**Supplemental Material**

Anna Ruth Pickett and Michelle L. Bell *

School of Forestry and Environmental Studies, Yale University, 195 Prospect St., New Haven,
CT 06511, USA; E-Mail: aruthpickett@gmail.com

***** Author to whom correspondence should be addressed; E-Mail: [michelle.bell@yale.edu](mailto:michelle.bell@yale.edu);
Tel.: +1-203-432-9869; Fax: +1-203-436-9135.

*Received: 5 October 2011; in revised form: 16 November 2011 / Accepted: 28 November 2011 /
Published:*

**Abstract:** Infants spend most of their indoor time at home; however, residential air quality is poorly understood. We investigated the air quality of infants’ homes in the New England area of the U.S. Participants (*N* = 53) were parents of infants (0–6 months) who completed telephone surveys to identify potential pollutant sources in their residence. Carbon monoxide (CO), carbon dioxide (CO_2_), particulate matter with aerodynamic diameter
≤0.5 µm (PM_0.5_), and total volatile organic compounds (TVOCs) were measured in
10 homes over 4–7 days, and levels were compared with health-based guidelines. Pollutant levels varied substantially across homes and within homes with overall levels for some homes up to 20 times higher than for other homes. Average levels were 0.85 ppm,
663.2 ppm, 18.7 µg/m^3^, and 1626 µg/m^3^ for CO, CO_2_, PM_0.5_, and TVOCs, respectively. CO_2_, TVOCs, and PM_0.5_ levels exceeded health-based indoor air quality guidelines. Survey results suggest that nursery renovations and related potential pollutant sources may be associated with differences in urbanicity, income, and presence of older children with respiratory ailments, which could potentially confound health studies. While there are no standards for indoor residential air quality, our findings suggest that additional research is needed to assess indoor pollution exposure for infants, which may be a vulnerable population.

**Keywords:** indoor air; infants; nurseries; carbon dioxide; carbon monoxide; volatile organic compounds; particulate matter

**Figure 1.** Hourly average CO values across time, by study subject. Dashed vertical lines divide days (*i.e.*, 12 midnight). The y-axis scales differ across figures.

**Figure 2.** Hourly average CO_2_ values across time, by study subject. Dashed vertical lines divide days (*i.e.*, 12 midnight). The y-axis scales differ across figures.

**Figure 3.** Hourly average PM_0.5_ values across time, by study subject. Dashed vertical lines divide days (*i.e.*, 12 midnight). The y-axis scales differ across figures.

**Figure 4.** Hourly average TVOC values across time, by study subject. Dashed vertical lines divide days (*i.e.*, 12 midnight). The y-axis scales differ across figures.

**Table 1.** Description of survey participants (*N* = 53), by monitoring group.

|  | **Number of participants (%)** | | |
| --- | --- | --- | --- |
|  | **All participants  (*N* = 53)** | **Monitoring participants  (*n* = 10)** | **Non-monitoring participants  (*n* = 43)** |
| **Annual household income** |  |  |  |
| <$10,000 | 1 (1.9%) | 1 (10%) | 0 (0%) |
| $10,000–20,000 | 0 (0%) | 0 (0%) | 0 (0%) |
| $20,000–50,000 | 10 (18.9%) | 2 (20%) | 8 (18.6%) |
| $50,000–75,000 | 8 (15.1%) | 1 (10%) | 7 (16.3%) |
| $75,000–100,000 | 12 (22.6%) | 3 (30%) | 9 (20.9%) |
| $100,000–150,000 | 12 (22.6%) | 3 (30%) | 9 (20.9%) |
| $150,000–200,000 | 4 (7.5%) | 0 (0%) | 4 (9.3%) |
| >$200,000 | 4 (7.5%) | 0 (0%) | 4 (9.3%) |
| Missing (declined to answer) | 2 (3.8%) | 0 (0%) | 2 (4.7%) |
| **Mother’s ethnicity** |  |  |  |
| Non-Hispanic White | 45 (84.9%) | 6 (60%) | 39 (90.7%) |
| Asian | 5 (9.4%) | 4 (40%) | 1 (2.3%) |
| Latina or Hispanic | 3 (5.7%) | 0 (0%) | 3 (7.0%) |
| **Mother’s education** |  |  |  |
| 13–14 years | 3 (5.7%) | 0 (0%) | 3 (7.0%) |
| 16 years | 17 (32.1%) | 3 (30%) | 14 (32.6%) |
| 17–19 years | 23 (32.1%) | 7 (70%) | 16 (37.2%) |
| >20 years | 10 (18.9%) | 0 (0%) | 10 (23.3%) |
| **Nature of birth** |  |  |  |
| Singleton birth | 51 (96%) | 9 (90%) | 42 (97.7%) |
| Twins | 1 (2%) | 1 (10%) | 0 (0%) |
| Triplets | 1 (2%) | 0 (0%) | 1 (2.3%) |
| Infant sex (*n* = 56) | (*n* = 56) | (*n* = 11) | (*n* = 45) |
| Male | 29 (51.8%) | 4 (36.4%) | 25 (55.6%) |
| Female | 27 (48.2%) | 7 (63.6%) | 20 (44.4%) |
| Infant age (*n* = 56) | (*n* = 56) | (*n* = 11) | (*n* = 45) |
| <31 days | 6 (10.7%) | 1 (9.1%) | 5 (11.1%) |
| 31–90 days | 19 (32.1%) | 5 (45.5%) | 13 (28.9%) |
| 91–150 days | 21 (37.5%) | 3 (27.3%) | 18 (40.0%) |
| >150 days | 11 (19.6%) | 2 (18.2%) | 9 (20.0%) |
| Infant in daycare | 5 (11.6%) | 0 (0%) | 5 (9.4%) |
| Hours in daycare (*n* = 5) | (*n* *=* 5) | (*n* *=* 0) | (*n* *=* 5) |
| 35 hours/week | 3 (60.0%) | n/a | 3 (60.0%) |
| 40 hours/week | 2 (40.0%) | n/a | 2 (40.0%) |
| **Participant’s relationship to infant** | | | |
| Mother | 46 (86.8%) | 8 (80%) | 38 (88.4%) |
| Father | 7 (13.2%) | 2 (20%) | 5 (11.6%) |

***Table 1.*** *Cont.*

|  | **Number of participants (%)** | | |
| --- | --- | --- | --- |
|  | **All participants  (*N* = 53)** | **Monitoring participants  (*n* = 10)** | **Non-monitoring participants  (*n* = 43)** |
| **Recruitment method** |  |  |  |
| Internet | 33 (62.3%) | 7 (70%) | 23 (60.4%) |
| Word of mouth | 13 (24.5%) | 3 (30%) | 10 (23.3%) |
| Flyer in hospital or baby store | 7 (13.2%) | 0 (0%) | 7 (16.3%) |
| Mother pregnant at time of survey | 0 (0%) | 0 (0%) | 0 (0%) |
| **Number of older siblings present in home** | |  |  |
| 0 | 40 (75%) | 8 (80%) | 32 (74.4%) |
| 1 | 13 (25%) | 3 (20%) | 11 (25.6%) |
| >2 | 0 (0%) | 0 (0%) | 0 (0%) |
| *Prevalence of health conditions in older siblings, of those with older siblings* (*n* = 13) | (*n* *=* 13) | (*n* *=* 2) | (*n* *=* 11) |
| Asthma | 2 (15.4%) | 0 (0%) | 2 (18.2%) |
| Allergies | 5 (38.5%) | 0 (0%) | 5 (45.5%) |
